# New Faces and Affiliations

**DOI:** 10.1155/S1064744996000488

**Published:** 1996

**Authors:** Sebastian Faro


Infectious Diseases in Obstetrics and Gynecology 4:255–256 (1996)
© 1997 Wiley-Liss, Inc.

### New Faces and Affiliations

It is with pleasure that I announce that our journal, *Infectious Diseases in Obstetrics and Gynecology*, has been designated as the official publication for the Infectious Disease Society of Obstetrics and Gynecology (IDSOG). The journal is proud and honored to be recognized as the official publication of this prestigious society.

After 4 years, the mission of the journal continues to be to publish the highest quality articles. These articles cover not only original research and case studies, but a timely review of subjects pertinent to the practice of infectious diseases in obstetric and gynecologic patients. Additionally, we have recently added a series designed to review and update our knowledge of antibiotic usage in obstetrics and gynecology. We also now publish the abstracts from the annual IDSOG scientific meeting in order to bring the readership the current scientific activities of the society. Most recently, we have also been selected to continue publishing both the abstracts and manuscripts from the proceedings of the International Group for Research in the Immunopathogenesis of Chlamydia Infections.

The journal looks foward to expanding while continuing to bring forth a variety of articles that are helpful to both the clinician and the investigator. Residents in training should find the journal helpful to their understanding of the basic principles of infectious diseases in obstetrics and gynecology. The broad scope and in-depth nature of our publication are designed to be most helpful to all physicians providing health care services to women. This includes not only ob/gyns, but general internists, family practitioners, pediatricians, and general surgeons as well.

In addition to the changes inside the journal, there have been some "behind the scene" changes as well. In September I assumed the position of Simpson Professor and Chair of the Department of Obstetrics and Gynecology at Rush-Presbyterian-St. Luke's Medical Center in Chicago, Illinois. When I made this move, I decided to bring the editorial office for this journal into my academic office and hired Donna Steinhagen to be my Editorial Associate. Donna brings to this journal the knowledge she gained as a developmental editor at Mosby-Year Book for the past 4 years. She has worked closely with a number of professionals in the field of women's health and is committed to this field. I feel the creation of an editorial team existing under one roof is necessary to keep up with the many changes that occur with expansion.

For your records our new address is:

*Infectious Diseases in Obstetrics and Gynecology*
1653 West Congress Parkway, 720 Pavilion
Chicago, IL 60612-3833
312-942-2188
312-942-3468 (fax)

Editorial

We encourage you to express your opinions to us on any subject. Feel free to submit questions regarding not only articles that appear in the journal, but those that relate to the significance of the practice of treating infectious diseases in obstetrics and gynecology. We are open to suggestions to improve the content of this journal and look forward to continued expansion. We promise to keep you posted!

*Sebastian Faro, M.D., Ph.D.*
*Editor-in-Chief*

